# Striving for humane deaths for laboratory mice: hypobaric hypoxia provides a potential alternative to carbon dioxide exposure

**DOI:** 10.1098/rspb.2022.2446

**Published:** 2023-04-26

**Authors:** J. M. Clarkson, J. E. Martin, J. Sparrey, M. C. Leach, D. E. F. McKeegan

**Affiliations:** ^1^ School of Biodiversity, One Health and Veterinary Medicine, College of Medical Veterinary and Life Sciences, University of Glasgow, Glasgow, UK; ^2^ School for Natural and Environmental Sciences, Newcastle University, Newcastle upon Tyne, UK; ^3^ Comparative Biology Centre, Newcastle University, Newcastle upon Tyne, UK; ^4^ The Royal (Dick) School of Veterinary Studies and The Roslin Institute, University of Edinburgh, Edinburgh, UK; ^5^ Livetec Systems Ltd, Wrest Park, Silsoe, Bedford, UK

**Keywords:** laboratory rodent, euthanasia, animal welfare, refinement, hypobaric hypoxia

## Abstract

Killing is often an unavoidable and necessary procedure for laboratory mice involved in scientific research, and providing a humane death is vital for public acceptance. Exposure to carbon dioxide (CO_2_) gas is the most widely used methodology despite well proven welfare concerns. Consequently, the continued use of CO_2_ and its globally permitted status in legislation and guidelines presents an ethical dilemma for users. We investigated whether killing with hypobaric hypoxia via gradual decompression was associated with better welfare outcomes for killing laboratory mice. We compared the spontaneous behaviour of mice exposed to CO_2_, decompression or sham conditions, and used analgesic or anxiolytic interventions to determine their relative welfare impact. Gradual decompression resulted in longer times to unconsciousness and death and the pharmacological interventions support the notion of a minimally negative animal experience, while providing further evidence for pain and anxiety associated with exposure to CO_2_. Decompression resulted in moderate ear haemorrhage, but our welfare assessment suggests this may happen when mice are unconscious. Hence, gradual decompression could be the basis of significant refinement for killing laboratory mice. Future work should corroborate behaviour with neurobiological markers of loss of consciousness to verify the conscious phase of concern for animal welfare.

## 1. Introduction

For rodents used for scientific purposes, killing is usually an unavoidable, necessary and legally mandated procedure upon completion of the scientific programme of work. Reflecting multiple ethical concerns, the use of animals in research is a prominent area of public debate and scientific reassurance that humane methods are used to end the lives of laboratory rodents is vital for public acceptance [1].

Exposure to carbon dioxide (CO_2_) gas in a rising concentration is favoured for killing laboratory rodents because of its capacity for high-throughput and non-contact nature [2]. However, there are significant welfare concerns surrounding its use, and several studies dispute its ability to provide a humane death [3]. Previous work has amply demonstrated a wide range of negative sensations and experiences that are likely to compromise mouse welfare such as pain, fear, anxiety, respiratory distress and dyspnoea [4–8], all of which occur during the conscious phase and at CO_2_ concentrations as low as 10% [9]. Mice actively choose to escape a chamber filling with CO_2_ at concentrations lower than those required to induce loss of consciousness [9]. Accordingly, the continued use of CO_2_ and its inclusion as a recommended method in existing guidelines and legislation (e.g. Schedule 1 of ASPA UK [10], EU Directive (2010/63/EU) [11], AVMA guidelines [12], ANZCCART [13]) presents an ethical dilemma for users and represents a risk to public perception and acceptance of *in vivo* biomedical research [3]. Urgent calls to reconsider the internationally accepted status of CO_2_ [3,4] have generated an unmet need to find an alternative high-throughput, non-contact killing method with better welfare outcomes. Mice remain the most widely used species for biomedical research with an estimated 5.5 million used in 2018 in Europe and Norway [14]. If a suitable alternative could be found, it would have the potential to have significant global impact, improving the welfare of millions of laboratory mice at the time of killing.

A candidate approach is hypobaric hypoxia, achieved via exposure to gradual decompression, which has received growing attention as a potential high welfare method of killing in both agricultural [15] and laboratory settings [16]. Gradual decompression describes a progressive reduction in atmospheric pressure, simulating ascent to a high altitude. High altitudes are associated with low total atmospheric pressures and partial pressures of atmospheric gases, including oxygen (O_2_), resulting in hypoxia [17]. Its application has already been validated for use as a high welfare method of stunning for poultry [15] resulting in its approval and inclusion in European Council Regulation (EC) No. 1099/2009 in 2018 [18]. Given the multiple anatomical, physiological and biological differences between mammals and birds, it is prudent to assume that responses to hypobaric hypoxia may differ. In previous work, we have demonstrated proof of principle for this technique in terminally anaesthetized mice, confirming feasibility while protecting animal welfare [16]. We systematically evaluated various decompression profiles, manipulating both the overall average decompression rate (75 m s^−1^ and 150 m s^−1^), and the degree of pressure change using stepwise phases to achieve accelerated, gradual and linear treatments. We showed that gradual decompression is a viable method for killing anaesthetized mice in non-prohibitive time frames, and with minimal pathological consequences. However, given mice were terminally anaesthetized, the full welfare impacts of hypobaric hypoxia for laboratory mice remain unknown. We did identify middle ear haemorrhage and congestion in unconscious mice [16], which could be associated with welfare harms upon application to conscious mice. Though it was unclear to what extent the observed pathological changes are associated with negative experiences and when these changes occurred (i.e. during decompression or recompression).

Behavioural assessment is essential for understanding the welfare state of an animal, providing information on health status and also the likely motivation for different activities [19]. As such, conducting in-depth behavioural observations during killing provides important insights into the likely affective state consequences of different death processes. Assessment should focus not only on key behaviours (or their cessation) indicative of loss of consciousness and death, but also on evidence of negative experiences and sensations such as pain, discomfort or anxiety. Previous work focusing on evaluating the welfare consequences of normobaric hypoxia (i.e. exposure to an inert gas) in laboratory rodents has demonstrated behavioural changes indicative of stress and/or anxiety [20–23]. Mice exposed to nitrogen (or CO_2_) elicit panic-like escape behaviours via vertical jumping or freezing behaviour, in addition to gasping indicative of dyspnoea [20–23]. In humans, self-reported symptoms during hypobaric hypoxia (in aviation training) primarily relate to barotrauma and include dysbarism of the ears and sinuses, trapped gas in the gastrointestinal tract and tooth pain [19]. Crucially, however, these are predominantly experienced during recompression (descent) [24], not decompression (ascent). Nonetheless, it is possible that related symptoms of physiological discomfort are caused in mice during decompression and must be fully explored.

The aim of this study was to determine the behavioural consequences of gradual decompression compared to CO_2_ in conscious mice and assess the likely welfare consequences of both methodologies through the application of analgesic and anxiolytic pharmacological intervention. We conducted a detailed assessment of spontaneous behaviours indicative of stress and anxiety, barotrauma and dyspnoea to improve our understanding of the welfare consequences of gradual decompression, informing us about its potential to provide a humane alternative to CO_2_. A pharmacological approach was adopted to further elucidate the animals’ likely experience during the conscious phase and facilitate interpretation of the likely causation of spontaneous behaviour. Despite the evidence that animals exposed to CO_2_ experience pain and distress, to our knowledge, few studies have used the application of pharmacological compounds such as analgesic and anxiolytic intervention in a welfare context for rodents [20,25–27]. By comparing hypobaric hypoxia with industry standard application of CO_2_, in addition to exploring the potential of hypobaric hypoxia, we also provide novel insights as to the likely experiences of mice exposed to CO_2_ killing.

## 2. Results

### (a) Terminal treatment effects on behavioural measures of hypoxia and death

**Figure 1.**
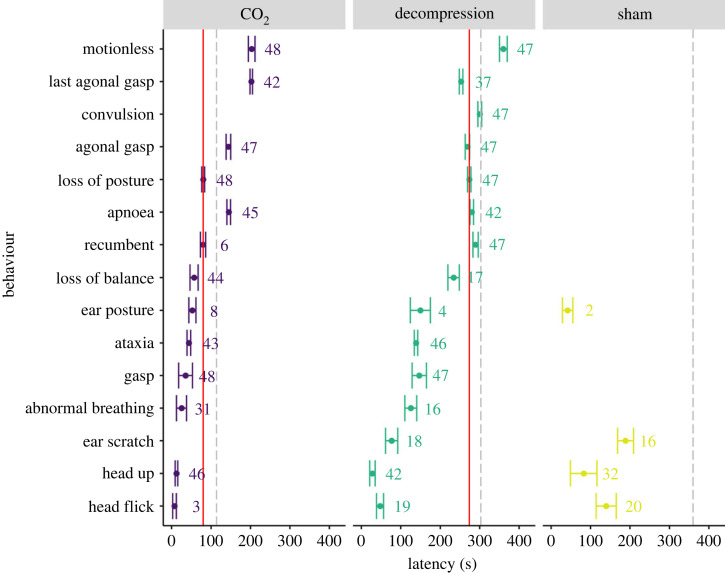
Mean (± s.e.) latencies (s) of key behavioural measures relating to respiratory responses (e.g. gasp, abnormal breathing, etc.), non-recovery indicators (e.g. motionless) and potentially negative sensations (e.g. ear scratch, head flick, etc.) according to terminal treatment (CO_2_ or decompression) or occurring during sham condition. The numbers immediately to the right of the s.e. bars represent the number of mice performing each behaviour out of a total *n* = 48 per treatment, *n* = 47 for decompression. The vertical solid red lines represent the mean latencies to reach loss of posture for each respective terminal treatment (CO_2_ 80.2 ± 3.76 s; decompression 273.7 ± 4.43 s) and was absent in the sham condition. The grey dashed lines represent the mean latencies to increase gas flow (CO_2_; left panel), reach target pressure of 200 mbar (end of phase 1 decompression; middle panel) or the completion of the 6-min sham (right panel) condition.

We compared the behavioural responses of laboratory mice to three experimental treatments: CO_2_ (top-fill procedure), gradual decompression and a sham condition. Irrespective of pharmacological intervention, we found general trends associated with the two terminal treatments. Behavioural events occurred earlier in CO_2_ compared to decompression due to the more gradual timescale of the decompression treatment resulting in longer cycle times. Behavioural latencies provide an overview of key response markers indicating death (e.g. cessation of breathing) and loss of consciousness (e.g. loss of posture (LOP)). On that basis, latencies to the exhibition of behaviours potentially indicative of negative experiences can be interpreted more confidently. Decompression resulted in longer latencies compared to CO_2_ for respiratory behaviours including: time to first gasp (*t*_18.1_ = 8.2, *p* < 0.0001), abnormal breathing (*t*_14.9_ = 7.8, *p* < 0.0001), apnoea (*t*_67.8_ = 36.2, *p* < 0.0001), agonal gasping (*t*_67.6_ = 23.2, *p* < 0.0001) and latency to last agonal gasp (*t*_68_ = 37.0, *p* < 0.0001) (figure 1). We also found longer latencies for decompression across behaviours indicative of hypoxia and death such as: time to ataxia (*t*_70.8_ = 15.8, *p* < 0.0001), loss of balance (LOB) (*t*_5.95_ = 10.3, *p* = 0.0001), LOP (*t*_16.6_ = 37.1, *p* < 0.0001), recumbency (*t*_43_ = 14.8, *p* < 0.0001) and motionless (*t*_18.7_ = 17.1, *p* < 0.0001). Only mice undergoing decompression convulsed and did so following LOP (figure 1).

Pharmacological intervention within each treatment did not affect the latency of most behaviours. However, we did observe some differences in the time to LOP according to drug intervention within each terminal treatment. Mice administered anxiolytic had reduced latencies to LOP compared to both saline control mice (approx. 33 s, *t*_14.6_ = 3.51, *p* = 0.0086) and analgesia treated mice (approx. 40 s, *t*_15.3_ = 4.66, *p* = 0.0008) when undergoing gradual decompression. By comparison, mice treated with analgesic intervention had shorter latencies to LOP compared to saline control mice (approx. 24 s, *t*_21_ = 2.69, *p* = 0.035) when undergoing terminal treatment with CO_2_ (see electronic supplementary material, S1).

Total duration of key behaviours is informative about an animal's likely experience, i.e. welfare state. Longer durations of several behaviours were observed in animals undergoing decompression compared to CO_2_, including time spent ataxic (135.6 ± 4.3 s versus 44.1 ± 4.1 s; *t*_112_ = 16.02, *p* < 0.0001), and time spent abnormally breathing (187.0 ± 12.6 s versus 124.0 ± 8.2 s; *t*_83_ = 5.11, *p* < 0.0001) with no effect of pharmacological intervention.

### (b) Behavioural indicators of potentially negative sensations

**Figure 2.**
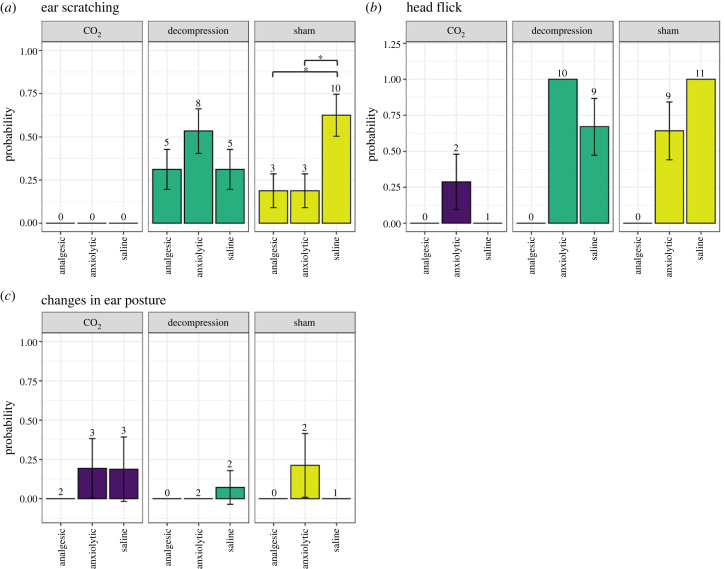
Mean (± s.e.) probability of a mouse performing each of the behaviours (*a*) ear scratching, (*b*) head flicks and (*c*) changes in ear posture for each terminal treatment (decompression, CO_2_) and sham conditions, with pharmacological interventions (analgesic, anxiolytic and saline). Numbers on the top of s.e. error bars denote the number of animals per group performing these behaviours out of a total *n* = 16. *p < 0.05, **p < 0.01, ***p < 0.001.

We observed several behaviours indicative of potentially negative sensations including ear scratching, changes in ear posture (a facial action unit of the mouse grimace scale [28]) and head flicking. However, these behaviours were not performed by all individuals undergoing decompression or CO_2_ and were also observed in mice exposed to sham conditions (figure 2; electronic supplementary material, S2). We found no effect of treatment (CO_2_, decompression or sham) on the likelihood of the occurrence of changes in ear posture (a facial grimace) (*X*_2(2)_ = 1.86, *p* = 0.395). However, the likelihood of occurrence for ear scratching (*X*_2(2)_ = 33.7, *p* < 0.001) and head flicking (*X*_2(2)_ = 11.7, *p* = 0.003) was reduced in animals exposed to CO_2_ compared to both sham and decompression, yet no difference in the likelihood of mice performing these behaviours between decompression or sham conditions. The likelihood of these behaviours occurring was not ameliorated by analgesic or anxiolytic intervention within the two terminal treatments but was in sham mice when administered either analgesia (odds ratio = 0.138, Z_ratio_ = 2.4, *p* = 0.043) or anxiolytic intervention (odds ratio = 0.138, *Z*_ratio_ = 2.4, *p* = 0.043) compared to mice administered saline (figure 2*a*).

**Figure 3.**
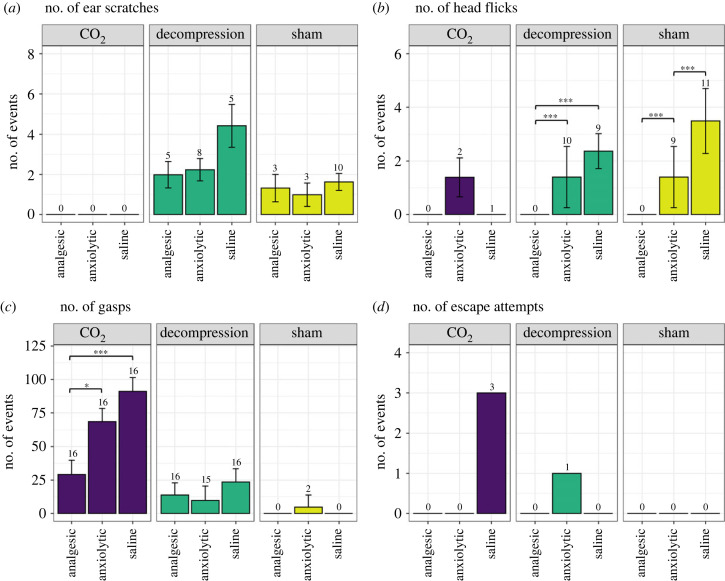
Mean (± s.e.) number of (*a*) ear scratches, (*b*) head flicks and (*c*) gasps, and raw data for frequency of (*d*) escape attempts, for each terminal treatment (decompression, CO_2_) and sham conditions treated with pharmacological interventions (analgesic, anxiolytic and saline). Numbers on the top denote the number of animals per group performing these behaviours out of a total *n* = 16. *p < 0.05, **p < 0.01, ***p < 0.001.

We also observed a relatively low frequency of events in those individuals performing these behaviours. We found no difference in the frequency of head flicks between decompression and sham mice, but did observe an effect in the frequency of ear scratches (*Χ*_2(1)_ = 9.95, *p* = 0.002). Mice exposed to decompression performed a greater frequency of ear scratches compared to sham mice overall (2.69 ± 0.44; 1.28 ± 0.357, *t*_24_ = 2.34, *p* = 0.028). This was irrespective of pharmacological intervention, with no effects on the frequency of ear scratching or head flicks overall or within decompression or sham treatments (figure 3*a*,*b*). Due to the low occurrence of ear scratching in animals exposed to CO_2_, meaningful statistical analyses were not possible. The same pattern of results was also observed when analysing the total duration of ear scratching.

Mice exposed to CO_2_ gasped more compared to both sham (*t*_26_ = 6.1, *p* < 0.0001; figure 3*c*) and decompression (*t*_31.3_ = 5.8, *p* < 0.0001), which did not differ from each other (*t*_22.9_ = 1.4, *p* = 0.362). Within the CO_2_ treatment, gasping was found to be affected by pharmacological intervention (*X*_2(4)_ = 14.83, *p* = 0.005), with analgesia treated mice gasping less compared to both saline (*t*_36_ = 4.2, *p* = 0.0006) and anxiolytic treated mice (*t*_34.9_ = 2.7, *p* = 0.0259). We observed more active escape attempts (vertical jumps) in mice exposed to CO_2_ (6.25%, *n* = 3/48) compared to decompression (2.1% *n* = 1/48) and sham (0%, *n* = 0/48) (figure 3*d*); however, due to low incidence and frequency, meaningful statistical analyses were not possible. Interestingly, all three individuals performing escape attempts in CO_2_ were saline treated mice (total escape attempts from 1 individual range from 1 to 6 occurrences).

**Figure 4.**
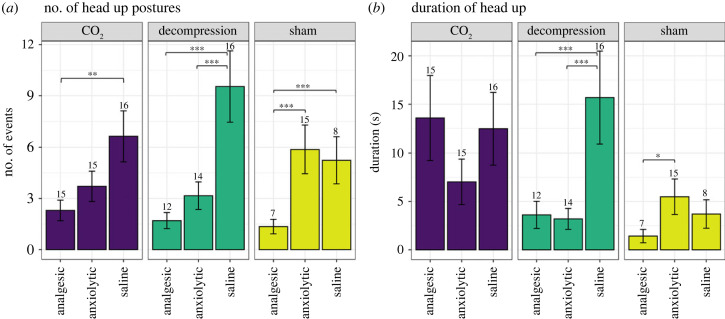
Mean (± s.e.) total (*a*) Number of head up postures and (*b*) Duration of time spent in a head up posture for each terminal treatment (decompression, CO_2_) and sham conditions treated with pharmacological interventions (analgesic, anxiolytic and saline). Numbers on the top of s.e. error bars denote the number of animals per group performing these behaviours out of a total *n* = 16. *p < 0.05, **p < 0.01, ***p < 0.001.

We assessed several postures considered to be potential indicators of negative sensations including changes to the position of the head, which were frequently observed. We found no difference in the frequency of head up postures across treatments (figure 4*a*); however, mice undergoing CO_2_ spent longer with their head in an upward position compared to those undergoing decompression (*t*_108_ = 2.69, *p* = 0.023) or sham (*t*_108_ = 3.73, *p* = 0.001), with no difference between decompression and sham mice (*t*_108_ = 1.75, *p* = 0.193) (figure 4*b*). Saline treated mice demonstrated more head up postures within both terminal treatments compared to anxiolytic and/or analgesic treated mice (CO_2_: analgesic versus saline *t*_1296_ = 3.25, *p* = 0.004; decompression: analgesic versus saline *t*_129_ = 5.17, *p* < 0.001, anxiolytic versus saline *t*_129_ = 3.44, *p* = 0.002); however, this was also true for mice undergoing the sham condition (analgesic versus saline *t*_129_ = 3.70, *p* < 0.001; figure 4*a*). In terms of total duration, intervention only had an effect within mice exposed to decompression, with mice administered saline spending more time with their heads up compared to analgesic or anxiolytic treated mice (analgesic versus saline *t*_108_ = 3.30, *p* = 0.0037, anxiolytic versus saline *t*_108_ = 3.80, *p* < 0.001; figure 4*b*).

### (c) The presence of typical species-specific behaviours

**Figure 5.**
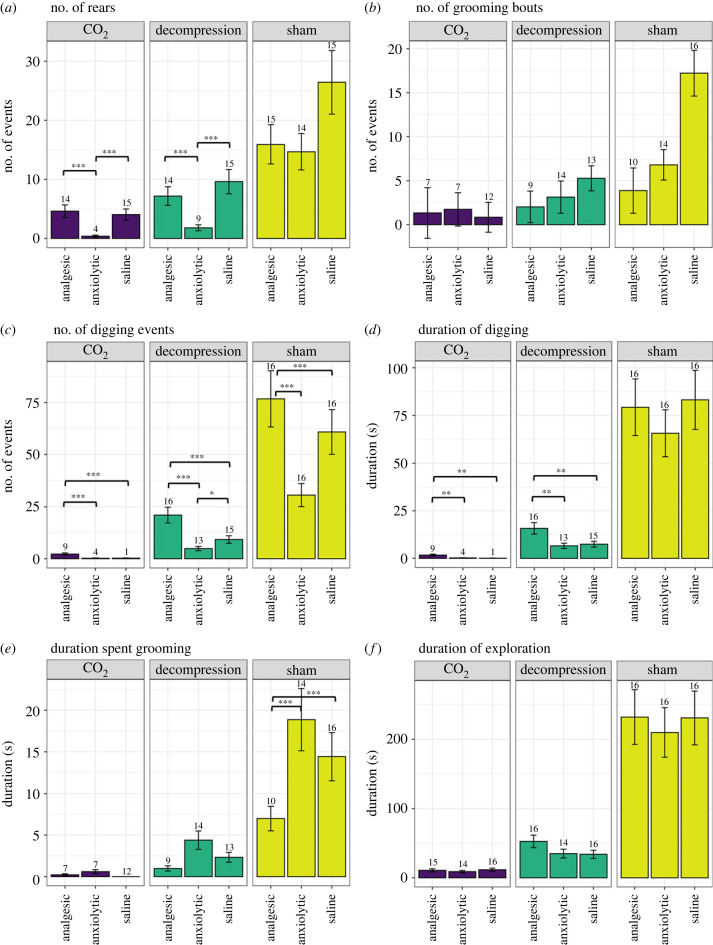
Mean (± s.e.) total (*a*) Number of rears, (*b*) Number of grooming bouts, (*c*) Number of digging events, (*d*) duration spent digging, (*e*) duration spent grooming and (*f*) duration spent exploring the periphery of the chamber, for each terminal treatment (decompression, CO_2_) and sham conditions treated with pharmacological interventions (analgesic, anxiolytic and saline). Numbers on the top of s.e. error bars denote the number of animals per group performing these behaviours out of a total *n* = 16. *p < 0.05, **p < 0.01, ***p < 0.001.

We explored a range of behaviours considered typical for the behavioural repertoire of laboratory mice (figure 5). Sham mice performed more rears (*X*_2(2)_ = 105.5, *p* < 0.0001), grooming bouts (*X*_2(2)_ = 27.4, *p* < 0.001) and digging events (*X*_2(2)_ = 315.0, *p* < 0.0001) compared to both terminal treatments (figure 5*a–c*). Mice exposed to decompression reared more (*t*_130_ = 4.12, *p* = 0.0002) and performed more digging events (*t*_130_ = 11.4, *p* < 0.0001) than mice exposed to CO_2_. This was also reflected in the duration data (figure 5*d*,*e*). Sham mice spent more time digging (*X*_2(2)_ = 276.7, *p* < 0.0001) compared to terminal treatments and spent longer grooming than mice undergoing decompression (*X*_2(3)_ = 51.4, *p* < 0.0001). Mice undergoing decompression spent more time digging than CO_2_ mice (*t*_130_ = 9.906, *p* < 0.0001). Sham mice spent more time exploring than mice in both terminal treatments (*X*_2(2)_ = 388.2, *p* < 0.0001), but again decompression mice spent more time compared to mice exposed to CO_2_ (*t*_130_ = 9.1, *p* < 0.0001). Mice administered analgesia performed more digging events and with greater duration compared to anxiolytic and saline treated mice (*X*_2(2)_ = 80.5, *p* < 0.0001, *X*_2(2)_ = 16.5, *p* < 0.0001 respectively), yet spent less time grooming than sham mice administered anxiolytic and saline (*t*_67_ = 5.2, *p* < 0.0001, *t*_67_ = 3.8, *p* < 0.0001 respectively).

### (d) Examination of the tympanic bullae

Given previous findings of minimal gross pathological damage across most organs including the lungs [16], we focused on the assessment of the tympanic membranes. The tympanic membranes were assessed by examination of the external ear canals with a stereomicroscope for mice exposed to CO_2_ and decompression. We found evidence of moderate haemorrhage of the tympanic bulla in mice undergoing decompression only (*n* = 47), with no evidence observed in mice exposed to CO_2_, although 100% of mice in both treatments had intact tympanic membranes.

## 3. Discussion

We report the behavioural consequences of gradual decompression in conscious laboratory mice in line with our aim to assess its potential to provide a humane alternative to killing by exposure to CO_2_. Collectively, our findings demonstrate that gradual decompression at a slow linear rate (approx. 40 m s^−1^ ascension equivalent) was associated with minimal negative behavioural indicators of pain and/or anxiety before loss of posture. Thus, decompression offers a promising avenue to viable and effective refinement.

We necessarily focused on behaviours indicative of the death process and on those that provide information on negative sensations such as pain and/or anxiety (i.e. indicative of welfare state). We took a mechanistic approach to ethogram construction to objectively describe behavioural actions without consequential descriptions that assume underlying motivational drive. Our evaluation was augmented by pharmacological interventions as an investigatory method to specifically ameliorate affective states of interest (pain and anxiety) and therefore we do not expect these to be used routinely in practice. Given the different modes of action and timescale of the killing methods being compared, in our welfare assessment we adopted an approach of comparing total counts and durations in the conscious phase, since these arguably represent total ‘harm’, whereas rate-based measures are less meaningful in terms of the overall animal experience. For example, five occurrences of a negative experience within a 10 s period would give a rate of 0.5 freq s^−1^, and the same rate would be achieved by 10 occurrences over a 20 s duration. However, the welfare insult to the animal in these two scenarios is different. This approach also prevents bias toward gradual decompression given the longer cycle times which results in more time available to express negative behaviours compared to CO_2_.

The primary welfare concern arising from our previous work on decompression was pain arising from suspected barotrauma in the middle/inner ear [16] and to mitigate this risk we applied a slower (linear) rate of gradual decompression here than in earlier work [16]. The rate we applied is unlikely to be associated with ear pain and/or discomfort in humans [29], but species-specific differences in barotrauma susceptibility are possible. We found evidence of moderate haemorrhage of the tympanic bulla in mice undergoing decompression, and consequently, specific attention was paid to evidence for head and ear related behaviours that might indicate ear pain and/or discomfort, and specifically whether they were mitigated by analgesic intervention. Although we found a greater likelihood of mice ear scratching and performing head flicks while undergoing decompression compared to CO_2_, these behaviours were equally likely to be observed in sham mice and as such we conclude that they are unlikely to reflect hypobaria. Further, we found no evidence to suggest a reduction in their occurrence with analgesic or anxiolytic intervention and therefore are unlikely to reflect pain and/or anxiety. Therefore, our findings suggest that the observed pathological consequences in the inner ear may be painless or are occurring after loss of consciousness, and we cannot rule out that they are occurring during recompression (descent) [24] not decompression (ascent). While we found no evidence of other validated pain behaviours in laboratory mice such as facial grimacing in either of the terminal treatments [28], the lack of facial grimacing in both groups is not surprising. It is well understood that validation of the Mouse Grimace Scale (MGS) is only sensitive in tested contexts with specific stimuli. Changes in the MGS are reported to be induced by acute pain with a duration of between 10 min and 24 h [28]. Further, studies focusing on evaluating pain in response to transient mild procedures (e.g. ear notching) have found no evidence of changes in the MGS [30]. Therefore, although we found a lack of facial grimacing, it was essential to examine potential changes in the MGS in both contexts given the differences in underlying mechanism of the terminal treatments (hypercapnic hypoxia versus hypobaric hypoxia). We observed a reduction in the duration of head up postures in analgesia and anxiolytic treated mice undergoing decompression compared to saline controls. However, saline treated mice performed more head up postures overall (irrespective of treatment), and despite the shorter cycle times, mice exposed to CO_2_ had longer total durations in a head up posture. Although the precise interpretation of this behaviour remains to be elucidated, it could represent an attempt to open the airways through stretching the neck to relieve dyspnoea [31]. Alternatively, it may reflect exploratory ‘environmental sampling’ behaviour, similar to mandibulation in poultry [15], which seems to be a response to hypoxia, not hypobaria [15,32]. Further, in the case of CO_2_ it could reflect an attempt to avoid gas accumulation at the bottom of the chamber compared to lower concentrations at the top of the chamber [33].

Dyspnoea reflects altered respiratory function and/or the activation of respiratory reflexes and is a negative affective experience in humans (commonly referred to as ‘air hunger’) [34]. Although neglected as an area of study in animals, air hunger has been highlighted as a significant animal welfare issue for animals killed via changes in atmospheric conditions, especially hypercapnia [23]. Although changes in respiratory sensation do not always have overt behavioural consequences, the degree of gasping is a useful proxy for evaluating the extent of respiratory challenge [3,23]. Mice undergoing CO_2_ killing gasped more than mice exposed to decompression and was mitigated by the provision of analgesia. This suggests that mice exposed to a hypercapnic environment experienced greater dyspnoea and possibly pain, supporting findings that hypercapnic environments increase respiratory drive more than inert hypoxia in humans [35]. Although we cannot fully rule out the potential effects of buprenorphine on respiratory sedation, we suspect this is unlikely given that it is usually associated with much higher doses in mice, e.g. >0.1 mg kg^−1^ [36], and we found no sedative effects in our sham mice when comparing to anxiolytic or saline treated mice. Hypercapnic stimulation of dyspnoea may also underpin the more active escape attempts observed among saline treated mice exposed to CO_2_ [34]. Vertical jumping is a well-documented behavioural response of mice exposed to CO_2_, which has previously been shown to be ameliorated by anxiolytic intervention [20]. We observed less jumping than previous work [20], possibly due to different methods of gas delivery. We employed best practice guidelines [4], and delivered gas to the top of the chamber. This was to ensure dissipation of the gas throughout the chamber given that CO_2_ is heavier than air, which if delivered to the bottom of the chamber, results in high concentrations accumulating at mouse level, and could explain the greater magnitude of jumping in previous work [20]. Indeed, it is possible that bottom fill protocols could expose mice to nociceptive concentrations of CO_2_ before loss of consciousness which could explain the more vigorous escape reactions reported in previous work. However, the purpose of our study was to determine whether hypobaric hypoxia could offer a humane alternative to CO_2_ when applied optimally. We demonstrate that mice perform more active escape attempts when exposed to optimally applied CO_2_ compared to the single mouse that performed a single vertical jump upon exposure to gradual decompression. Therefore, results demonstrating any refinement to optimal ‘gold standard’ CO_2_ application are likely to be extrapolated to larger refinements for suboptimal CO_2_ application.

Given the gradual nature of the decompression profile we applied, loss of posture and time to death were extended compared to CO_2_. Nevertheless, both terminal treatments resulted in a consistent and predictable sequence of behavioural events, albeit occurring at later timepoints for decompression. An important consideration therefore is the reduced time available for mice undergoing CO_2_ killing to perform behaviours (including species typical behaviours) so that direct comparison of behavioural events and durations between the two terminal treatments must be made cautiously. Given the different mechanisms of action (hypercapnic hypoxia versus hypobaric hypoxia) and induction times, the assessment of species typical behaviour was important when considering gradual decompression to ensure the prolonged induction time was not associated with prolonged suffering. Importantly, mice undergoing decompression spent time performing species-specific exploratory and maintenance behaviours (e.g. grooming) during the longer induction time. This strongly suggests that this period is minimally unpleasant for mice, as we would expect these species typical behaviours to be overtaken by pain, anxiety or stress related behaviours during an aversive situation. Hypoxia is generally considered aversive for mice; however, this is based on findings from work focused on normobaric hypoxia [20–23] with ill-defined and/or poorly controlled oxygen availability, and the mechanisms underpinning hypobaric hypoxia are somewhat different [37]. It is possible that the slow linear rate of decompression employed here represents hypoxic exposure, which is gradual enough to be insidious, avoiding significant negative consequences before unconsciousness.

We utilized well-validated analgesic and anxiolytic compounds that have been previously studied in laboratory mice [38–41] and employed meaningful control groups (sham and saline treatment groups). However, we cannot exclude potential side effects on spontaneous behaviour due to sedative or excitatory effects. Although there was evidence of some sedative and excitatory effects of diazepam and buprenorphine on a few behaviours, e.g. rearing and digging respectively in sham mice, these were minimal, and the overall duration of exploration, for example, was unaffected. Instead, our work highlights the importance of developing detailed species-specific ethograms focused on highly detailed categorization of behaviours, and meaningful control groups to allow disentanglement of treatment effects from potential side effects, enhancing interpretation.

## 4. Conclusion

Gradual decompression was associated with fewer behavioural indicators of pain and anxiety in mice than those elicited by currently recommended CO_2_ killing practices. The replacement of CO_2_ with a practical, high-throughput and humane alternative has been identified as an urgent requirement in recent years [3]. Our robust experimental design utilizing pharmacological intervention and a sham treatment reveals the potential of gradual decompression to fulfil this unmet need. Our findings provide encouraging behavioural evidence of reduced welfare harm during gradual decompression compared to hypercapnia. A comprehensive investigation of the potential of gradual decompression as a euthanasia method for mice should also corroborate time to loss of posture with neurobiologically relevant markers of loss consciousness to verify the conscious phase of concern. Ideally, operant techniques could also be employed to determine the animal's degree of active aversion, compared to CO_2_, before loss of consciousness.

## 5. Methods and materials

### (a) Animals, housing and husbandry

Based on *a priori* power analysis from similar work conducted in pigs [42], 144 male C57BL/6J mice were obtained from Charles River UK with Specific Pathogen Free (SPF) health status in accordance with FELASA health monitoring recommendations. This resulted in 16 mice in each experimental group which consisted of a 3 × 3 factorial design including three experimental treatments (CO_2_, decompression or sham) and three drug intervention treatments (analgesia, anxiolytic or saline). Mice were delivered at approximately eight weeks of age across two batches (arrival dates; batch 1: 20 April 2021; batch 2: 11 May 2021). Mice were socially housed in triplets in filter top caging (1284L, Techniplast) with aspen sawdust bedding, nesting substrate (sizzle nest, Datesand Ltd), a clear Perspex tunnel (Datesand Ltd) with food (BK001, Special Diet Services) and tap water available ad libitum. Mice were maintained on a 12 : 12 light dark cycle (07:00 on, 19:00 off) under constant temperature and humidity in line with UK Home Office code of practice guidelines. All animals were acclimatized for one week to both the laboratory and handling practices prior to commencement of experimental work and weighed 24.4 ± 0.14 g (min: 20.1 g; max: 28.6 g) at the time of experimental work. All mice were handled using refined tunnel handling techniques in line with local refined handling practices to mitigate against background stress and anxiety [43]. All animals were checked daily, and no adverse effects were reported.

### (b) Acclimatization

Prior to the experiment and during the acclimatization week, all mice were acclimatized to home cage transport to the experimental room, non-aversive tunnel handling and exposure to the testing box. Between the hours of 13:00 and 16:00 animals were removed from their home cage via refined handling techniques and placed into the testing box across three consecutive sessions (commencing three days prior to terminal treatment application), with duration of exposure to the testing box (described below) increasing on subsequent exposures (1 min, 3 min and 6min exposures). In line with rodent holding rooms, the experimental room was kept under constant temperature (21 ± 4°C) and humidity (55 ± 10%) in line with code of practice guidelines.

### (c) Apparatus

**Figure 6.**
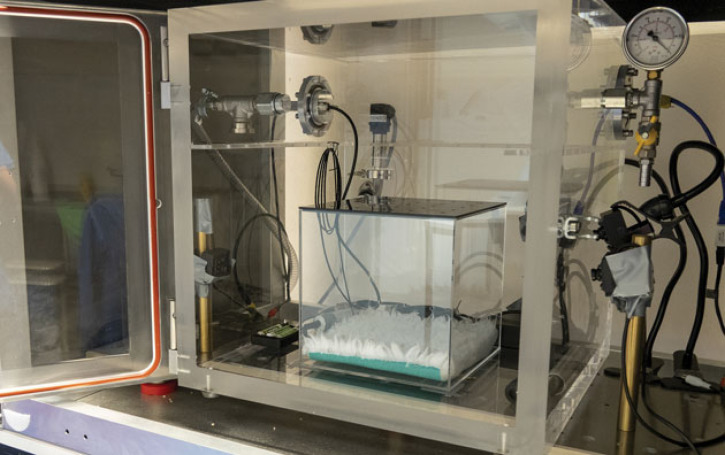
Photograph of experimental setup including chamber consisting of outer 40 × 40 × 40 cm (L, W, H; inner dimensions) acrylic chamber and inner Perspex 20 × 20 × 20 cm (L, W, H; inner dimensions) box with Vetbed. Multiple wide-angle cameras located on three sides of the chamber allowed for full bilateral cranial–caudal view of the mouse and facilitated complete behavioural observations. Additional sensors including pressure, temperature, relative humidity and carbon dioxide sensors, as well as a microphone for ultrasonic vocalization analysis were also present.

A dual-purpose chamber was custom designed (Livetec Systems Ltd) and made from transparent acrylic (30 mm thick) with external dimensions including attachments of 610 mm (W) × 610 mm (D) × 470 mm (H) and internal dimensions of 400 mm (W) × 400 mm (D) × 400 mm (H) (figure 6). The chamber allowed application of both exposure to a gas (i.e. CO_2_) and implementation of hypobaric hypoxia (gradual decompression), with a hose connected to a medical grade CO_2_ cylinder (201-D, BOC, UK) and a central dual tap hose splitter to facilitate gas delivery or gradual decompression. To achieve decompression, the chamber connects to an automated programmable logic controller (PLC) system from which fully flexible programming of parameters is controlled to achieve a target decompression profile using a touch screen user interface. The chamber is connected to a vacuum pump (DVP lubricated rotary vane vacuum pump, LC25 2018) by a hose and a proportional control valve enables fixed decompression rates, adjustable according to cycle time or pressure thresholds. Chamber pressure (mbar) was monitored and recorded every second using precision probes during baseline, decompression and recompression phases of the cycles. Following each cycle, recompression was facilitated manually over a period of at least 3 min (225 ± 2.94 s) by an air admittance valve, monitored with a vacuum gauge. The pressure change during recompression was monitored and maintained by placement of a marker on the air admittance valve to ensure consistent recompression between individuals and was timed and recorded using a stopwatch. This ensured consistent, slow recompression at an overall average rate that was no faster than a descent equivalent to 65 m s^−1^.

**Figure 7.**
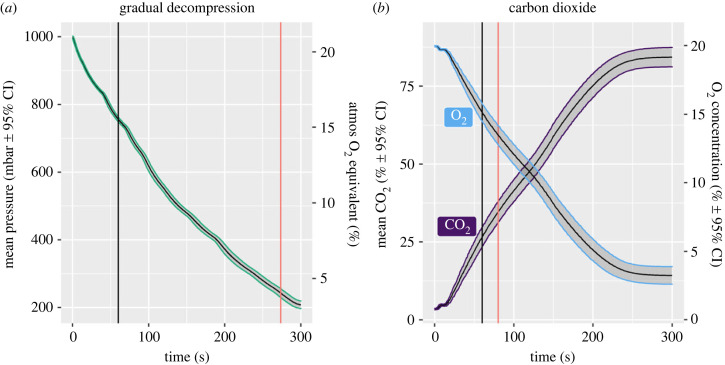
(*a*) Gradual decompression treatment profile: mean (±95% CIs) pressure (mbar) for mice undergoing gradual decompression during the first phase to reach the target pressure of 200 mbar. This was followed by a 5-min hold period until death. Secondary axis represents calculated atmospheric oxygen equivalent (%) at sea level (1013 mbar). (*b*) Exposure to carbon dioxide (CO_2_) treatment profile: mean (±95% CIs) CO_2_ concentration (%) (purple; left axis) and oxygen levels (%) (blue; right axis) for mice undergoing carbon dioxide terminal treatment. Black vertical line represents 60 second marker and the red line denotes the mean latency to reach LOP for each respective terminal treatment.

For exposure to CO_2_, the chamber was connected to a second PLC which was programmed to record the levels of CO_2_ and O_2_ gas every second. Both O_2_ and CO_2_ levels were monitored within the chamber at mouse height for each individual (figure 7). The chamber was also fitted with multiple sensors to allow the continuous recording (every 1 s) of temperature (°C) and relative humidity (%).

### (d) Pharmacological interventions

Mice were randomly assigned to one of three drug intervention groups via random number generator (random.org): analgesic, anxiolytic or saline, blocked by cage to ensure intervention was balanced across cages (i.e. one of each intervention per cage). Drug interventions were administered subcutaneously (s/c) in the scruff forty minutes prior to exposure to the experimental treatment to ensure sufficient time for pharmacokinetic efficacy of the active compounds [38,39]. Buprenorphine (analgesic) was administered s/c at a dose of 0.05 mg kg^−1^ [38], diazepam (anxiolytic) was administered s/c at a dose of 2.5 mg kg^−1^ [40,41] and the volume administered (including saline control, Baxter Healthcare, 0.9% sodium chloride injection, USP) was balanced across compounds according to the animal's bodyweight (0.0067 ml g^−1^) in line with LASA good practice guidelines (allowing up to 0.02 ml g^−1^) [44]. Exact injection time was recorded to ensure consistent timing between individuals and pharmacokinetic efficacy of the compounds prior to experimental treatment exposure.

### (e) Exposure to experimental terminal treatments

This study involved the exposure of mice to one of three experimental treatments: CO_2_ (top-fill protocol [4]), gradual decompression and a sham condition (where mice were placed in the chamber but no terminal treatment was applied). According to a balanced, randomized-block factorial design using a Latin square, mice were exposed to their designated experimental treatment individually. Blocking factors included batch and cage, to ensure all three animals undergoing the terminal treatments were killed on the same day and to ensure all mice undergoing the sham treatment were from the same cage to reduce stress and mitigate against social isolation upon their return [45]. This design was fully balanced with respect to experimental treatment and drug intervention across batches. Three animals underwent each experimental treatment (decompression, CO_2_ or sham) per day consisting of each of the three drug interventions (analgesic, anxiolytic or sham), resulting in 9 animals killed per day across 8 consecutive days. Terminal trials were conducted at the same time of day throughout between the hours of 8:00 and 13:00. Mice were removed from their home cage via refined handling techniques and were placed in the testing chamber. Clean Vetbed was placed for each individual to mitigate against potential crossover of odour cues [46]. Following a 30 s baseline period, animals were exposed to the experimental treatment.

### (f) Gradual decompression

In line with previous work [15,16], the applied decompression profile consisted of two distinct phases. Phase 1 involved decompression from sea level (1013 mbar) to a target pressure of 200 mbar (approx. 11 800 m equivalent altitude) followed by a subsequent hold phase (Phase 2) for a minimum of 5 min and confirmation of death (i.e. >30 s motionless). The rate of decompression was relatively consistent, with the 200 mbar pressure target (Phase 1) reached in 303 s (289–332 s), resulting in an average 38.9 m s^−1^ (35.5–40.8 m s^−1^) equivalent rate of ascension to 11 800 m. One animal undergoing gradual decompression was excluded as the rate achieved was not representative of the target approximately 40 m s^−1^ treatment (achieved rate of ascension 27.8 m s^−1^), due to a PLC error. Decompression rate was based on previous findings where faster rates of decompression (75 m s^−1^ and 150 m s^−1^) were associated with potential concern for barotrauma of the inner ear and why a slower rate was employed (approx. 40 m s^−1^) [16]. The mean total cycle time (Phases 1 and 2) was 607 s (590–680 s).

### (g) Carbon dioxide

In line with current best practice guidelines [4], CO_2_ was applied at a flow rate of approximately 20% of the chamber volume per minute, which was introduced to the top of the chamber, mediated manually via a flowmeter. Gas flow was maintained at 20% volume per minute until loss of posture (LOP) (mean: 114 s; range: 90–180 s), at which point it was then increased to 80% of the chamber volume per minute until observed cessation of breathing (mean: 224 s; range: 199–314 s). Upon respiratory arrest, a subsequent two-minute dwell time ensued resulting in a mean total cycle time of 346 s (319–434 s).

### (h) Sham treatment

The sham treatment consisted of placing the mice in the chamber without initiating terminal treatments (CO_2_ or gradual decompression). The sham treatment was designed to match a conservative estimate of the duration of the conscious phase during gradual decompression (6 min). Following exposure to the chamber for this period, mice were returned to their home cage and kept alive at the establishment.

### (i) Pathology observations

Following CO_2_ and gradual decompression terminal treatments only and upon removal from the chamber, death was secondarily confirmed in accordance with Schedule 1 through permanent cessation of the circulation by severing the femoral artery for all mice. Tympanic membranes were assessed by examination of the external ear canals with a stereomicroscope by a trained examiner. The tympanic membranes were recorded as intact or ruptured for all mice undergoing CO_2_ and gradual decompression terminal treatments (*n* = 16 per terminal treatment + intervention combination).

### (j) Behavioural observations

The behaviour of each mouse was video recorded using a GeoVision surveillance system (GV800B) connected to four wired cameras (Bird box camera 1080p with IR Night vision) sitting at different positions outside of the decompression chamber, providing a full bilateral cranial–caudal view. The system allowed direct live monitoring of the mouse on an external monitor and captured footage for future analysis. Behavioural footage for each mouse was continuously observed using Noldus Observer XT (v. 12) by a single blinded observer. An ethogram (outlined in electronic supplementary material, table S3) was developed in line with previous work on gaseous killing methods in laboratory mice [22,47,48]. We took a mechanistic approach to ethogram design to avoid presumptions of motivational drive and minimize observer interpretation of intention. Behavioural variables measured included latencies, counts and total durations. Data were exported from Noldus Observer to Microsoft Excel.

### (k) Statistical analyses

Statistical analyses were conducted in R (v. 4.0.3, R Core Team) via the R Studio platform (v. 1.3.1093, RStudio, PBC, 2009–2020). All data were collated and processed within R using the tidyverse package [49]. All graphical summaries were created using the ggplot2 package [50]. Behavioural data were analysed with generalized linear mixed models (GLMMs) via the glmmTMB package [51] and general linear mixed models (GLMs) via the lme4 package [52] and were used to identify fixed effects which may affect each behavioural measure. Model fit was determined by examination of residuals via the DHARMa package [53] and appropriate error distributions set for GLMMs. Binary variables such as whether an individual ear scratched (yes/no) were analysed with the family link set to binomial. Subset analyses were performed on infrequent behaviours including counts for ear scratches and head flicks to determine the relationship of fixed effects on the likelihood of occurrence, only across animals that performed them. All models included fixed effects of treatment (3 levels: decompression, CO_2_ or sham), intervention (3 levels: analgesic, anxiolytic or saline). Interactions between fixed effects were included in the model. Relative humidity and temperature were included in the model as covariates to ensure they did not have a significant effect upon outcome variables and removed if non-significant to improve model fit. All models included the covariate mouse weight (accounting for weight differences) and random nested effects, with unique cage number nested within batch to account for non-independence of mice from the same cage. Statistical significance based on *p* < 0.05 threshold was calculated using the ANOVA function (car package) to ascertain differences derived from fixed effects and interactions, and only statistically significant results are reported. Pairwise comparisons were reported using estimated marginal means via the emmeans package [54], with *p* values adjusted for multiple comparisons using the Tukey method.

## Data Availability

All data generated in and analysed during the current study are publicly available in the University of Glasgow's research online data repository (http://dx.doi.org/10.5525/gla.researchdata.1415) [56]. The data are provided in electronic supplementary material [57].
